# Metabolite Profiling of Macroalgae: Biosynthesis and Beneficial Biological Properties of Active Compounds

**DOI:** 10.3390/md22100478

**Published:** 2024-10-19

**Authors:** Maria Carpena, Cláudia S. G. P. Pereira, Aurora Silva, Paula Barciela, A. Olivia S. Jorge, Ana Perez-Vazquez, Antia G. Pereira, João C. M. Barreira, M. Beatriz P. P. Oliveira, Miguel A. Prieto

**Affiliations:** 1 Department of Analytical Chemistry and Food Science, Instituto de Agroecoloxía e Alimentación (IAA)—CITEXVI, Universidade de Vigo, Nutrition and Bromatology Group, 36310 Vigo, Spain; mcarpena@uvigo.es (M.C.); mass@isep.ipp.pt (A.S.); paula.barciela@uvigo.es (P.B.); anolijorge@gmail.com (A.O.S.J.); ana.perez.vazquez@uvigo.es (A.P.-V.); antia.gonzalez.pereira@uvigo.es (A.G.P.); 2 LAQV/REQUIMTE, Department of Chemical Sciences, Faculdade de Farmácia, Universidade do Porto, R. Jorge Viterbo Ferreira 228, 4050-313 Porto, Portugal; claudia.guimaraes.pp@gmail.com (C.S.G.P.P.); beatoliv@ff.up.pt (M.B.P.P.O.); 3 LAQV/REQUIMTE, Instituto Superior de Engenharia do Porto, Instituto Politécnico do Porto, Rua Dr. António Bernardino de Almeida 431, 4249-015 Porto, Portugal; 4 Investigaciones Agroalimentarias Research Group, Galicia Sur Health Research Institute (IIS Galicia Sur), SERGAS-UVIGO, 36312 Vigo, Spain; 5 Centro de Investigação de Montanha (CIMO), Instituto Politécnico de Bragança, Campus de Santa Apolónia, 5300-253 Bragança, Portugal; jbarreira@ipb.pt; 6 Laboratório Associado para a Sustentabilidade e Tecnologia em Regiões de Montanha (SusTEC), Instituto Politécnico de Bragança, Campus de Santa Apolónia, 5300-253 Bragança, Portugal

**Keywords:** macroalgae, metabolites, phlorotannins, bromophenols, pigments, biological properties

## Abstract

Macroalgae are known as abundant sources of phytochemicals, which offer a plethora of beneficial biological properties. Besides being the most notable classes of compounds found in macroalgae, phlorotannins, bromophenols, and terpenoids comprise some of the most relevant for their biological properties. Phlorotannins, mainly prevalent in brown algae and structurally characterized as complex polyphenolic compounds derived from phloroglucinol units, possess robust antioxidant, anti-inflammatory, antitumor, and cytotoxic activities, modulated by factors such as the degree of polymerization and environmental conditions. Bromophenols, halogenated compounds found in algae and other marine organisms, exhibit significant antioxidant and antiviral properties. Their diverse structures and bromination patterns contribute to their potential as therapeutic and chemical defense agents. Pigments (chemically described as primary terpenoids) play a critical role in light absorption and energy transfer in macroalgae and are divided into three main groups: (i) carotenoids, which are primarily found in brown algae and provide photoprotective and antioxidant benefits; (ii) chlorophylls, known for facilitating the conversion of light into biological energy; and (iii) phycobilins, which are mostly found in red algae and play important roles in light absorption and energy transfer, besides providing remarkable health benefits. Finally, secondary terpenoids, which are particularly abundant in red algae (e.g., the Rhodomelaceae family) are central to cellular interactions and exhibit significant antioxidant, antimicrobial, antidiabetic, and anti-inflammatory properties. This study represents a detailed analysis of the biosynthesis, structural diversity, and biological activities of these macroalgae metabolites, emphasizing their potential biological properties.

## 1. Introduction

Macroalgae are marine organisms that represent an integral element of marine ecology. These organisms are found all over the world’s coasts and are thought to include over 164,000 species. Of these, approximately 9800 species are categorized as seaweeds, with only 0.17% having been domesticated for commercial purposes [1]. They can be classified into three primary taxonomic groups based on their pigmentation: Chlorophyta (green algae), comprising 6851 living species; Phaeophyta (brown algae), with 2124 identified species; and Rhodophyta (red algae), which includes approximately 7276 species [2,3]. Macroalgae have sophisticated biosynthetic pathways that can generate an array of metabolites crucial for their survival and adaptability, besides possessing distinctive and advantageous biological properties [4]. The development of metabolomic analysis has improved our understanding of these complex pathways, providing detailed insights into the metabolic systems and the bioactive compounds they produce [5].

Primary metabolism is a vital biogeochemical pathway that is directly involved in cell development and proliferation [6]. The most important primary metabolites include proteins, lipids, and carbohydrates. Proteins are present in algae as grouped amino acids or in conjugated forms such as phycobiliprotein or glycoproteins [7] and could be of economic importance as sources of bioactive peptides [8]. Moreover, mycosporine-like amino acids (MAAs) are attractive molecules with photoprotective and antioxidant functions [9]. Polysaccharides, which function as energy storage and/or structural molecules, are usually present as complex molecules such as alginate, laminarin, carrageenan, or ulvan (simple sugars might also be found) [10,11]. Lipids, which include fats, oils, phospholipids and steroids, are also present in quantities of up to 5% in dry weight [12]. Together, these metabolic products enable cells to multiply, divide, and continue to perform their vital functions.

On the other hand, metabolites present in minor concentrations are essential to macroalgae’s biology, allowing them to survive in their frequently harsh (due to exposure to ultraviolet UV radiation, variations in salinity and temperature, and pollution) maritime conditions [13]. These substances have a variety of adaptive and defensive uses, such as the ability of macroalgae pigments to participate in photosynthesis and their protective action against the effects of ultraviolet (UV) radiation on cellular structures [14], or the capacity of phlorotannins to protect against numerous external factors (e.g., light, nutrient scarcity, salinity, ultraviolet radiation, or dryness) [15]. These phlorotannins, which are found exclusively in the brown algae family, are particularly noteworthy compared to the other polyphenols produced by algae due to their unique biochemical properties. Phlorotannins are biosynthesized at the cellular level via the acetate–malonate pathway within the Golgi apparatus. They are essential for the structural integrity of algae, particularly in response to oxidative stress within the cell wall [15].

Bromophenols are another important type of metabolite that will receive special attention in this review, particularly due to their interesting biological properties. These compounds are highly widespread, occurring more expressively in Rhodophyta species, but also in brown and green macroalgae [16]. In either case, they are biosynthesized in the presence of bromoperoxidases, hydrogen peroxide, and bromide; structurally, bromophenols have one or several benzene groups and different degrees of bromination and hydroxylation [17].

Terpenoids or terpenes are lipophilic metabolites derived from isoprene units and are classified into different types depending on the number of these units, such as hemi-, mono-, sesqui-, and diterpenoids [18]. In brown algae, terpenoids are a significant part of the metabolites, particularly in species from the *Sargassaceae* and *Dictyotaceae* families, which account for over 80% of these compounds [19]. Among terpenes, pigments stand out due to their bioactivity [14,20] and their role in defining an important factor to categorize algae as red (phycobilins), green (chlorophylls), or brown (carotenoids) species [21]. Among carotenoids, astaxanthin, lutein, fucoxanthin, canthaxanthin, zeaxanthin, and β-cryptoxanthin are important examples. Due to the presence of double bonds in their structure, carotenoids are effective in providing various health benefits and protecting other molecules from the oxidative stress caused by active radicals through multiple mechanisms [14].

Previous reviews on macroalgae metabolites focus on macronutrients (proteins, lipids, and carbohydrates); there are also some reports on polyphenols, halogenated compounds, or terpenoids [12], but are limited to their extraction, identification technologies, and potential applications [22,23]. Other works cover micro- and macroalgae, delving into their bioactive properties, but lacking enough attention to the chemical structures of the responsible compounds [24]; in turn, when the association among structural characterization and biological activity is attained, only a particular activity type is covered [25]. 

This review aims to fill these gaps by focusing on the biosynthesis, structural diversity, and biological activities of metabolites in macroalgae. It is characterized by linking biosynthetic pathways with the bioactivity of specific compounds, providing a comprehensive view of their potential applications. The articles selected for this review prioritize recent peer-reviewed studies examining the biosynthesis and activity of metabolites from macroalgae. This ensures the inclusion of the most relevant and up-to-date findings, meeting to the needs of researchers and industry stakeholders.

## 2. Phlorotannins

### 2.1. Biosynthesis & Structural Classes of Phlorotannins

Phlorotannins are widely distributed among marine organisms, particularly in brown algae like kelps, rockweeds, and species within the *Sargassaceae* family [26]. These compounds can account for up to one-sixth of the dry weight of *Phaeophyceae*, depending on the species [27], while the red algae content of phlorotannins ranges between 1.8 and 3.2% [28]. Phlorotannins may exist freely or form complexes with various constituents of cellular walls, such as alginic acid [27], due to their hydrophilic properties, which facilitates firm binding to biopolymers, such as carbohydrates and proteins, as well as to divalent metals ions [29,30]. As a result, the highest concentrations of phlorotannins in brown algae are typically located in the meristematic or reproductive sections of the thallus [31].

Structurally, phlorotannins resemble terrestrial plant tannins, as they are produced by polymerizing phloroglucinol (1,3,5-trihydroxybenzene) monomer units, in a similar way to the biosynthesis of tannins in terrestrial plants from alcohol monomers [32]. Phlorotannins represent a diverse group of complex polyphenolic compounds, consisting of different levels of polymerization of phloroglucinol units, with a wide variety of chemical structures [29,33]. The establishment of a taxonomy system for marine phlorotannins was first based on the configuration and bonding patterns of phloroglucinol monomers, the incorporation of an extra hydroxyl group in fuhalols, the detection of carmalols, and the potential occurrence of halogens or sulfate groups [32].

**Figure 1:**
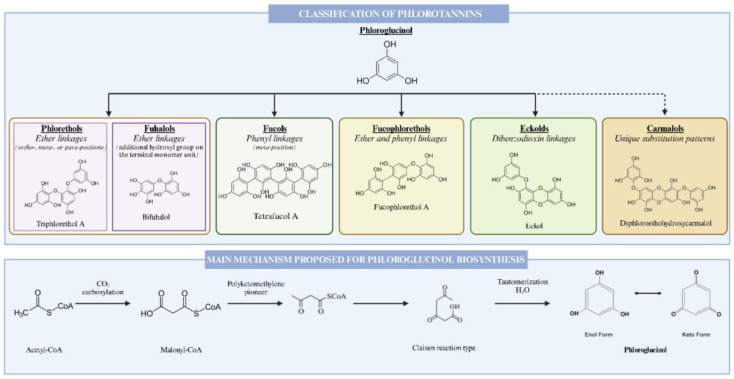
An overview of the classification and biosynthesis of phlorotannins in macroalgae.

Generally, phlorotannins are categorized into four categories based on their linkage types. The subclasses include compounds with ether linkages (fuhalols and phlorethols), those featuring phenyl linkages (fucols), substances with both ether and phenyl linkages (fucophlorethols), and the ones characterized by a dibenzodioxin linkage (eckols). In particular, fuhalols and phlorethols, found in species such as *Fucus vesiculosus* and *Fucus spiralis*, are known for their antioxidant properties. Fucols, which have phenyl linkages, are present in *Sargassum* spp. and contribute to their ecological role. The fucophlorethols, characterized by both ether and phenyl linkages, are found in *Fucus serratus*. Finally, the eckols, known for their dibenzodioxin linkage, are mainly present in *Ecklonia cava*, which exhibits various bioactive properties (Figure 1) [26,34].

Fucols consist of phlorotannins with inter-phloroglucinol linkages in the meta-position, as exemplified by compounds like tetrafucol A, tetrafucol B, and heptafucol [35]. Phlorethols, on the other hand, are composed of phloroglucinol residues that are connected via ether bonds in ortho-, meta-, or para-positions, illustrated by triphlorethol A, tetraphlorethol C, and tetraphlorethol B [36]. Fuhalols and eckols possess an additional hydroxyl group on the terminal monomer unit, like trifuhalol A, bifuhalol, and trifuhalol B [37], whereas eckols feature 1,4-dibenzodioxin in their structure, such as eckol, dieckol, and 2-phloroeckol [38]. Fucophloroethols combine ether and phenyl bonds, as seen in fucophlorethol B, fucophlorethol A, and fucodiphlorethol B [39]. Additionally, a new class of phlorotannins, termed carmalols, has been proposed, comprising a distinct set of phlorethols with unique substitution patterns compared to eckols, such as diphloroethohydroxycarmalol [39].

The structural diversity of phlorotannins, along with the possibility of multiple bonding positions between monomers at the same molecular weight, results in numerous structural and conformational isomers [40]. Moreover, phlorotannins exhibit a broad spectrum of molecular weights, influenced by various factors such as edaphoclimatic conditions, intraspecific variations (i.e., species, size, tissue, and age), and the method of extraction, with the predominant molecular weight falling between 10 and 100 kDa, but reaching as much as 650 kDa [41].

Hence, while the occurrence of phlorotannins in brown algae is universally recognized, comprehensive studies detailing the characterization of these intricate polymeric structures are notably scarce. Indeed, various methodologies have been explored to elucidate the structure of phlorotannins in their natural state [42]. Significant variations in both the molecular structure and the polymerization degree of polyphenols have been observed, even among closely linked species of brown algae [43]. Consequently, the molecular profiles of phlorotannins exhibit a high level of complexity and are highly specific to individual species [43]. For instance, *Sargassum muticum* from the North Atlantic coasts alone exhibited up to 53 types of phlorotannins from the fuhalol and phlorethols classes [42].

The biochemical process underlying the synthesis of phlorotannins remains inadequately elucidated, with various theories proposed, encompassing the condensation of acetate and malonate units, as well as the involvement of the shikimate or phenylpropanoid pathways [44,45]. Phlorotannins may also exist in sulfated or halogenated forms, and their synthesis occurs (as previously indicated) within the Golgi apparatus through an acetate–malonate pathway, similarly referred to as the polyketide pathway [44,45]. This pathway encompasses the conversion of two molecules of acetyl-CoA and carbon dioxide into malonyl-CoA. Subsequently, the polyketomethylene pioneer, produced through the assembly of three malonyl-CoA units, undergoes a cyclization reaction akin to the “Claisen type”, resulting in the constitution of a hexacyclic ring system. This stems from their diverse bioactivities, comprising antioxidant, anti-inflammatory, antimicrobial, antiallergic, antitumor, angiogenesis, and tyrosinase inhibitory properties [26]. Consequently, brown seaweeds are recognized as a valuable source of nutritious food, owing to the health benefits related with phlorotannins [45]. Phlorotannins derived from brown seaweeds exhibit low toxicity across various biological systems, including cell lines, invertebrates, microalgae, and animals such as fish, mice, rats, dogs, and humans. However, the evaluation of their safety and toxicity, specifically in aquaculture fish, livestock, and companion animals, remains limited. Further investigations are warranted to comprehensively assess the safety and toxicity of phlorotannins in these species. Such research could elucidate the potential applications of phlorotannins as functional foods, animal feeds, and pharmaceuticals [36].

The medicinal effectiveness of phlorotannins is intricately tied to their chemical structure, with the degree of polymerization emerging as a significant determinant. Generally, oligo-phenols are considered more bioactive than highly polymerized molecules [46]. Furthermore, the bioactivities of phlorotannins can vary according to the harvesting time and geographic conditions. Seasonal variations affect the concentration of phlorotannins, with the peak polyphenolic content typically observed in summer [31]. A summary of the primary bioactivities associated with phlorotannins present in algae will be provided in the following sections.

### 2.2. Biological Properties of Phlorotannins with Relevance in Disease Treatment

#### 2.2.1. Antioxidant Activity of Phlorotannins

Phlorotannins, known for their ability to donate electrons due to their electron-donating groups, participate in efficient electron donation reactions, yielding phenoxyl radical intermediates that are stabilized via resonance delocalization in the presence of oxidizing agents [47]. Various investigations have underscored the robust antioxidant attributes of phlorotannins derived from algae against oxidative damage caused by free radicals. This antioxidant efficacy may arise from the specific scavenging of radicals generated during peroxidation, the scavenging of oxygen-containing molecules, or their metal-chelating abilities [45].

Numerous studies (Table 1) have employed the reduction of reactive oxygen species (ROS) as the primary parameter to assess the antioxidant activity of phlorotannins. This evaluation typically involves scavenging assays utilizing 1,1-diphenyl-1,2-picrylhydrazyl (DPPH), 2,2′-azinobis (3-ethylbenzothiazoline-6-sulphonic acid) (ABTS), peroxyl, alkyl, hydroxyl radical (•OH), and superoxide anion ($O_{2}^{\cdot -}$) scavenging activities [48]. Comparative analyses have revealed that phlorotannins often exhibit stronger antioxidant effects in contrast to well-established antioxidants, such as ascorbic acid [26,49,50,51], catechin, α-tocopherol [26,49], and butylated hydroxytoluene (BHT) [48], highlighting their remarkable antioxidant activity. Similar trends were observed for $O_{2}^{\cdot -}$ scavenging, with significantly lower half maximal inhibitory concentration (IC_50_) values than resveratrol, ascorbic acid, or α-tocopherol (IC_50_ = 21, 16, and 12 µM, respectively) [49] (Table 1).

Regarding structure–activity relationships, Li et al. (2009) anticipated that the number of hydroxyl groups in phlorotannins influences their antioxidant activity [52]. Additionally, researchers have suggested that phlorotannins with molecular weights within the 8–18 kDa range exhibit the highest antioxidant activity, with higher molecular weights showing decreased activity, possibly due to mutual shielding effects via hydrogen bond formation around their reducing centers [53].

#### 2.2.2. Antimicrobial Properties of Phlorotannins

Phlorotannins have also been highlighted as antimicrobials. Their activity, higher against Gram-positive bacteria has been related to their ability to alter cell membranes and disrupt cell walls [21]. Nagayama et al. (2002) assessed the antimicrobial properties of phlorotannins extracted from *Ecklonia kurome* against a variety of foodborne pathogens, including different methicillin-resistant *Staphylococcus aureus* (MRSA) strains and *Streptococcus pyogenes*. Their findings revealed that phlorotannins were particularly effective against MRSA and showed the highest efficacy against *Campylobacter* species. Specifically, the minimum bactericidal concentrations for the phlorotannins, dieckol, and 8,8′-bieckol against *Campylobacter jejuni* were determined to be 50 mg/L, 0.03 μmol/mL, and 0.03 μmol/mL, respectively [54]. Antifungal and antiviral activities have also been linked to phlorotannins. For example, phloroglucinol, eckol, 7-phloroeckol, phlorofucofuroeckol, and dieckol extracted from *Ecklonia cava* showed inhibitory effects against the Influenza A virus, by inhibiting neuraminidase [55].

#### 2.2.3. Anti-Inflammatory Properties of Phlorotannins

In addition to their antioxidant properties, phlorotannins are involved in various anti-inflammatory processes. These compounds can inhibit the expression of pro-inflammatory cytokines, regulate the activity and/or expression of essential enzymes, and impede transcriptional regulation. Due to these properties, phlorotannins are considered promising candidates for therapeutic applications to reduce inflammation and treat inflammation-related ailments, including tumors [48]. It has been observed that phlorotannins can inhibit the lipopolysaccharide (LPS)-induced production of NO and prostaglandin E2 (PGE2) by downregulating the expression of NO synthase and cyclooxygenase-2 (COX-2) in LPS-induced RAW 264.7 murine macrophages [39]. Additionally, they decrease the levels of pro-inflammatory cytokines, like interleukin 1 β (IL-1β), interleukin 6 (IL-6), or tumor necrosis factor α (TNF-α); inhibit inflammatory mediators (iNOS and COX-2) and transcription factors (NF-κB, the nuclear translocation of p50 and p65 subunits, and AP-1); and activate Akt and p38 MAPK signaling pathways [56]. In vivo studies show that phlorotannins exhibit anti-inflammatory properties through multiple pathways, including the regulation of COX-2, hyaluronidases (HAases), intercellular adhesion molecule-1 (ICAM-1), IL-1β, IL-6, iNOS, mitogen-activated protein kinases (MAPKs), TNF-α, tumor necrosis factor receptor-associated factor 6 (TNFR), and Toll-like receptors 2/4/7 (TLR-2/4/7) [15,57]. Furthermore, it has been demonstrated that phlorotannins can mitigate the “cytokine storm” caused by viral infections, which can lead to a sharp increase in cytokine levels in the blood and trigger autoimmune attacks on the body’s own cells and tissues [58].

Metabolic disorders, like insulin resistance and cardiovascular disease, are closely linked to chronic inflammation [59]. Studies investigating crude phlorotannins have revealed their extensive and intricate anti-inflammatory effects, attributed to the synergistic action of various phlorotannins [60]. However, several studies suggest that the main phlorotannins responsible for this bioactivity in *Ecklonia stolonifera* are phlorofucofuroeckol A and B, 6,6′-bieckol, 2-phloroeckol, and 974-B [56,61]. More examples can be seen in Table 1. Moreover, some studies indicate that the anti-inflammatory properties of certain phlorotannins are superior to those of reference drugs. For instance, the IC_50_ values for the inhibitory activity against the HAase of phlorotannins from *E. bicyclis* and *E. kurome* were 30, 35, 180, 90, and 140 μg/mL, for catechin, epigallocatechin gallate, and disodium cromoglycate, respectively, suggesting the strong potential of phlorotannins as anti-HAase drugs [62].

#### 2.2.4. Antitumor and Cytotoxic Activity of Phlorotannins

Phlorotannins have shown to be effective radical scavengers, suggesting their potential indirect use in reducing cancer formation in the human body [45]. However, various studies indicate that the antitumor capacity of phlorotannins is more complex. These compounds have exhibited antiproliferative, antimetastatic, and antiangiogenic effects in diverse kinds of cancer [48]. For example, the phlorotannin dioxinodehydroeckol, isolated from *Ecklonia cava*, has been shown to diminish the proliferation of human breast cancer cells by inducing apoptosis [63]. Other phlorotannin derivatives, such as phlorofucofuroeckol G, dieckol, eckol, and phlorofucofurofuroeckol from *E. cava*, have demonstrated strong cytotoxic effects on human cancer cell lines, like HeLa (human cervical cancer cells), HT1080 (human fibrosarcoma cells), A549 (human lung carcinoma cells), and HT-29 (human colon adenocarcinoma cells), while being less cytotoxic to MRC-5s (normal human lung fibroblasts) [64]. More examples of antitumor activity can be observed in Table 1. Additionally, various studies indicate that the phlorotannin content is directly proportional to the antitumor activity. For instance, samples of *Sargassum muticum* that were collected in Norway (nearly 5 mg phlorotannins/g of extract) exhibited growth inhibitions nearly twice as strong as algae from Portugal (approximately 4 mg phlorotannins/g of extract) [42].

Regarding the mechanism of action of phlorotannins, the primary mechanism responsible for their antiproliferative action occurs by interacting with multiple mediators of the apoptotic signaling cascade, thereby initiating cell death through the induction of apoptosis [65]. In this respect, the phlorotannins that have been shown to be most efficient in inducing apoptosis include dieckol, phloroglucinol, phlorofucofuroeckol A, dioxinodehydroeckol, and eckol [15]. Other mechanisms of action of phlorotannins include inhibiting angiogenesis and invasion (dicromeol, phloroglucinol); inducing drug resistance in cancer stem cells (phloroglucinol, eckol); and activating innate and adaptive immune responses (eckol) [15].

#### 2.2.5. UV-Absorbing Activity

Brown algae are characterized by having a higher tolerance to UVB irradiation compared to other algae, due to their concentration of phlorotannins. Therefore, in recent decades, there have been endeavors to develop formulations that are rich in phlorotannins, with the potential to reduce skin photodamage [31]. Examples include the phlorotannins dieckol and eckol, isolated from *E. cava*, which have been demonstrated to decrease intracellular ROS generated by γ radiation [66], as well as to protect the skin against radiation-induced cellular DNA damage and membrane lipid peroxidation [45]. Other studies have demonstrated that dieckol at concentrations of 100 μM can increase cell survival up to 77.1% in UVB-irradiated human dermal fibroblasts [67]. Another study, using the same concentration, showed that this extract can increase cell survival in human epithelial keratinocytes by 88.42% [68].

Furthermore, lower concentrations of dieckol (50 μM) have been shown to reduce UVB-induced DNA damage by 57.8% [67], also yielding significant results in alternate animal model systems like zebrafish [68]. Diphlorethohydroxycarmalol, extracted from *Ishige okamurae,* has been investigated for its photoprotective properties in human dermal fibroblasts cells that were exposed to UVB radiation. Results indicate that, at concentrations of 250 μM, this compound can scavenge 45.57% of ROS and reduce DNA damage by 49.33% [69]. Therefore, phlorotannins have great potential to be used as functional constituents in pharmaceutical and cosmeceutical products for skin treatment [45].

**Table 1:** Macroalgal phlorotannins properties and activity results.

Properties	Active Compound	Species	Assay	Results (μg/mL)	Ref.
Antioxidant	974-A and 974-B	Ecklonia kurome (B)	DPPH	IC50 = 2.4/2.6	[26]
Antioxidant	Dieckol	Ecklonia cava (B)	HOO	IC50 = 3.5	[52]
Antioxidant	Phlorotannin extract	Fucus vesiculosus (B)	DPPH	IC50 = 3.8–4.7	[50]
		Ascophylum nodosum (B)		IC50 = 6.3–7.7	
Antioxidant	Eckstolonol, dieckol, and phlorofucofuroeckol A	Ecklonia stolonifera (B)	DPPH	IC50 = 2.1/1.5/1.1	[51]
Antioxidant	Eckol, phlorofucofuroeckol A, dieckol and 8,8′-bieckol	Eisenia bicyclis, Ecklonia cava, and Ecklonia kurome (B)	DPPH	IC50 = 6.2, 2.9/3.1/3.5	[49]
			$O_{2}^{\cdot -}$	IC50 = 2.5/1.9/1.8/1.5	
Anti-inflammatory	Phlorotannin extract	Fucus distichus (B)	PMA-stimulated RAW 264.7 cells	IC50 = 37	[57]
Anti-inflammatory	Phlorotannin extract	Eisenia bicyclis and Ecklonia kurome (B)	Inhibitory activity against hyaluronidase	IC50 = 30/35	[62]
Anti-inflammatory	Dieckol, eckol, phlorofucofuroeckol A, and phlorofucofuroeckol B	Ecklonia stolonifera (B)	Inhibited LPS-induced NO and PGE2	IC50 = 72 and 98	[56]
	Phlorofucofuroeckol isomers A and B		Inhibition of NO production	IC50 = 1.7/2.9	
Anti-inflammatory	Dieckol, eckol, and 7-phloroeckol	Eisenia bicyclis (B)	LPS-induced NO production in RAW 264.7 cells	IC50 = 51.42/52.86/26.87	[70]
Anti-inflammatory	Phlorotannin-purified extracts	A. nodosum and Alaria esculenta (B)	CaCo-2 cell line	IC50 = 33/7	[71]
Antitumor	Phloroglucinol derivate	Ecklonia cava (B)	MCF-7 cell line	IC50 = 2.4	[63]
	Dioxinodehydroeckol			IC50 = 24	
Antitumor	Dieckol	Ecklonia cava (B)	A2780 and SKOV3 cells	IC50 = 84.3/99.6	[72]
Antitumor	Phlorotannin extracts	Laminaria japonica (B)	BEL-7402 and murine leukemic cells	IC50 = 200/120	[73]
Antitumor	Phlorethols	Costaria costata (B)	Inhibitor of the α-NaGalase of cancer cells	IC50 = 15.2/5.7	[74]
Antitumor	Phlorotannin-purified fractions	Fucus vesiculosus (B)	MKN-28, Caco-2, and HT-29 cell lines	IC50 = 56.3/97.4/118.8	[75]

Abbreviations: DPPH: 2,2-diphenyl-1-picryl-hydrazyl radical; $O_{2}^{\cdot -}$: superoxide-radical-scavenging activity; IC_50_: half maximal inhibitory concentration; PMA: phorbol 12-myristate 13-acetate; RAW 264.7: murine macrophage cell line; BEL-7402: human hepatocellular carcinoma cell line; MCF-7: human breast cancer cell line; A2780: ovarian cancer cell line; SKOV3: ovarian cancer cell line with epithelial-like morphology; LPS: lipopolysaccharide; NO: nitric oxide; PGE2: prostaglandin; E2; CaCo-2: human colon adenocarcinoma-derived cell line; MKN-28: human gastric carcinoma cell line; HT-29: human colon adenocarcinoma-derived cell line; HOO˙: peroxyl-radical-scavenging activity. (G): green macroalgae; (R): red macroalgae; (B): brown macroalgae.

## 3. Bromophenols

### 3.1. Biosynthesis & Structural Characterization of Bromophenols

**Figure 2:**
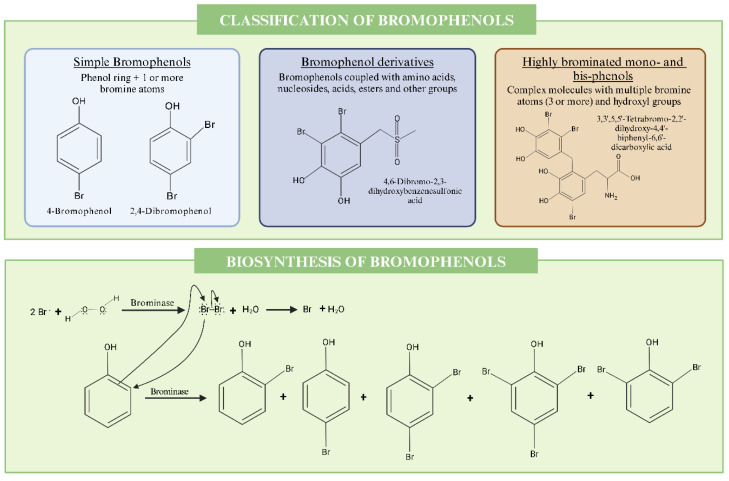
An overview of the classification and the biosynthesis of bromophenols from algae.

Bromophenols are found in a wide range of marine organisms, like algae, fish, and prawns [76], and exhibit diverse structural classes, which are primarily influenced by their bromination patterns and the presence of different functional groups. A commonly applied classification system defines three main structural classes: simple bromophenols, bromophenol derivatives, and highly brominated mono- and bis-phenols. Simple bromophenols are usually found in *Rhodomela confervoides* and *Gracilaria* spp. Bromophenol derivatives are derived from *Chondrus crispus*, while highly brominated mono- and bis-phenols are identified in *Pterocladiella capillacea* and *Asparagopsis taxiformis* (Figure 2).

Simple bromophenols, as the name suggests, are basic structures consisting of a phenol ring with one or more bromine atoms. In these simple phenols, the structure and properties are significantly affected by intramolecular hydrogen bonding and the electrostatic effects of bromine substituents. For instance, increasing the number of bromine atoms connected to the phenol ring causes the O-H bond length to increase and the C-O bond length to decrease, causing a red shift in the O-H stretching frequency along with a blue shift in the O-H torsional frequency [76]. Increasing bromination also increases the steric hindrance in the molecule, which is a determinant of its stability, besides being associated with lower pK_a_ values (higher acidity) [76]. It is worth noting that these bromophenols are not the most common in biological organisms as they are usually man-made for industrial use, mainly as a flame retardant. In recent years, worries have arisen due to the formation of toxic brominated dioxins as combustion products [77].

Bromophenol derivatives are complex structures that include conjugates with amino acids, nucleosides, carboxylic acids, esters, and other groups. *Rhodomela confervoides*, for instance, produces bromophenols coupled with pyroglutamic acid derivatives and one bromophenol coupled with deoxyguanosine [78]. In this same alga, bromophenols can be found coupled with nucleoside-based derivatives and brominated tetrahydroisoquinolines [79]. Likewise, bromophenol derivatives, incorporating cyclopropane moieties, have been artificially synthesized as inhibitors of carbonic anhydrase enzymes, showing potent inhibitory effects in the nanomolar range against human cytosolic isoenzymes, highlighting their potential therapeutic applications [80]. In addition, bromophenols with sulfur and nitrogen groups have demonstrated antifungal activity and have been identified in various algae, including *Symphyocladia latiuscula* [81].

Highly brominated mono- and bis-phenols are complex molecules with multiple bromine atoms (three or more) and hydroxyl groups. These are often associated with a high radical-scavenging capability and antioxidant activity and are considered of possibly great therapeutical value. Examples include the aldose reductase inhibitors 2,2′,3,6,6′-pentabromo-3′,4,4′,5-tetrahydroxydibenzyl ether, or 2,2′,3,5′,6-pentabromo-3′,4,4′,5-tetrahydroxydiphenylmethane (among others), isolated from the alga *Symphyocladia latiuscula.* [82].

There are several pathways that biological organisms can use to produce bromophenols. Marine bacteria, for instance, utilize brominase enzymes that perform decarboxylative halogenation, while fungi perform a laccase-catalyzed oxidation [83,84]. However, here, we will focus on the biosynthesis of bromophenols by macroalgae.

In marine algae, bromoperoxidase enzymes play a crucial role. These enzymes convert precursors like phenol, 4-hydroxybenzoic acid, and 4-hydroxybenzyl alcohol into bromophenols [17]. The enzyme activity and bromophenol content in these algae show significant seasonal variations, reaching maximal values in the summer months [17]. Bromoperoxidase enzymes catalyze the halogenation of organic compounds by transferring bromine ions to phenolic substrates. This enzyme uses hydrogen peroxide (H_2_O_2_) as an oxidizing agent to facilitate the bromination reaction [17]. In the case of *Ulva lactuca*, the bromoperoxidase enzymes utilize phenol, 4-hydroxybenzoic acid, and 4-hydroxybenzyl alcohol to produce several bromophenols [17]. It is important to note that the enzyme’s activity can vary significantly, depending on environmental factors and the algae’s physiological state. The biological role of bromophenols has been widely discussed, and some roles have been proposed, such as antioxidant protection, antimicrobial protection, chemical defense, and chemical signaling between algae [85]. The involvement of bromophenols in chemical defense systems may occur in several ways. Firstly, owing their antimicrobial activity, bromophenols can inhibit the growth of various marine bacteria and fungi, which helps to protect algae from microbial infections and biofouling [86].This antimicrobial activity ensures that algae can survive and thrive in competitive marine environments by warding off harmful microorganisms. Bromophenols are thought to help protect against herbivores. Research indicates that these compounds contribute to the distinct sea-like taste and flavor of marine organisms, which may discourage predators [87]. In addition, it has been theorized that bromophenols, due to their antioxidant properties, might also play a role in signaling oxidative stress levels within algal cells, modulating the community’s response to environmental stressors like UV radiation or pollution, and promoting the overall integrity of the organism [87].

### 3.2. Biological Properties of Bromophenols with Relevance in Disease Treatment

#### 3.2.1. Antioxidant Activity of Bromophenols

Algae-derived bromophenols are recognized for their potent antioxidant activities. These compounds act as effective radical scavengers due to their unique structures, which often include multiple bromine and hydroxyl groups [16,88]. Free radicals play a crucial role in the development of several health disorders, including cancer, diabetes, neurologic, metabolic, and inflammatory diseases. Combating these radicals with antioxidant compounds may have a beneficial effect on human health, as it may prevent free-radical damage and associated diseases [88,89].

There are two proposed chemical mechanisms to explain the antioxidant activity of bromophenols: (1) the H-atom transfer, where a radical gains a hydrogen atom from the antioxidant, converting the antioxidant into a radical, and (2) the electron transfer, where the antioxidant donates an electron to the radical, forming a radical anion [85]. The antioxidant activity of specific bromophenols is highly dependent on their structure and group substitutions. For example, electron-donating groups at *ortho* and *para* positions relative to the phenolic OH group enhance antioxidant activity by lowering the bond dissociation energies. Furthermore, the stability of radicals formed via the H-atom transfer mechanism is influenced by the ability to delocalize the unpaired electron across the molecule, particularly involving bromine atoms and the aromatic ring [85].

*Vertebrata lanosa* is an example of red alga with a high amount of antioxidant bromophenols. Among these, 2,2′,3-tribromo-3′,4,4′,5-tetrahydroxy-6′-hydroxymethyldiphenylmethane is highlighted due to its high antioxidant activity, outperforming the natural antioxidants luteolin and quercetin in cellular essays [85]. The antioxidant activity is also strong in nitrogen-containing bromophenols, such as those isolated from another marine red alga, *Rhodomela confervoides*, which showed potential to adjuvate specific therapies [90].

#### 3.2.2. Antiviral Activity of Bromophenols

Two bromophenols isolated from the red alga *Polysiphonia morrowii*, 3-bromo-4,5-dihydroxybenzyl methyl ether and 3-bromo-4,5-dihydroxybenzaldehyde, showed significant in vitro antiviral capacities against two fish pathogenic viruses: infectious hematopoietic necrosis virus (HNV) and infectious pancreatic necrosis virus (IPNV). This activity might be due to the interaction of those bromophenols with viral particles or cellular components to inhibit viral entry or replication. Accordingly, there is a potential use of these bromophenols as natural antiviral agents in fish farming, which could lead to the development of new antiviral drugs or health-promoting feed additives for aquaculture [91].

#### 3.2.3. Other Activities (Anti-Inflammatory Activity, Antibacterial, Antidiabetic, Anti-Obesity, and Enzyme Inhibition) of Bromophenols

Red algae bromophenols, such as those isolated from *Rhodomelaceae* species, demonstrate a strong inhibition towards the enzyme glucose-6-phosphate dehydrogenase (G6PH), a key enzyme in the pentose phosphate pathway and crucial for producing NADPH, which is necessary for fatty acid and cholesterol biosynthesis [92]. This enzyme is suggested as a target for therapies against diseases related to lipid metabolism, such as obesity and certain cancers. Dimeric bromophenols are better at inhibiting G6PH than their monomer counterparts [92].

Likewise, marine algae have been used for a long time as a folk remedy against diabetes [93]. This is echoed in scientific findings that suggest the antidiabetic activity of several bromophenol isolates from algae, such as in the case of the bromophenol derivatives from the red alga *Rhodomela confervoides*, which showed a strong inhibition of protein-tyrosine phosphatase 1B (PTP1B)’s activity, a protein that regulates the insulin signaling pathway. Extracts from the same red alga also showed the ability to decrease the blood glucose level in diabetic rats [94]. α-Glucosidase is another enzyme that plays a role in diabetes, breaking down complex carbohydrates into glucose, which is then absorbed into the bloodstream. Several isolated bromophenols have shown inhibitory activity against α-glucosidase, with the corresponding IC_50_ values decreasing with the increase of bromination in the bromophenols [95].

Several studies have pointed to the possible anti-cancer activity of marine bromophenols, such as 3-bromo-4,5-dihydroxy benzoic acid methyl ester and 3-bromo-4,5-dihydroxy-benzaldehyde (isolated from *R. confervoides*), which showed selective cytotoxicity against several cancer cell lines: KB (human oral epidermoid carcinoma cells), Bel 7402 (human liver cancer cells), and A549 [96]. In addition, some bromophenols isolated from the brown algae *Leathesia nana* also showed cytotoxic activity against several human cancer cell lines, including A549, BGC-823 (human stomach cancer cells), MCF-7 (human breast cancer cells), Bel 7402, and HCT-8 (human colon cancer cells) [97].

Bromophenol derivatives were also studied for their antibacterial properties. Researchers synthesized a series of compounds by incorporating bromophenolic and phenolic groups into their molecular structure using N-bromosuccinimide in acetonitrile at 50 °C. The synthesized bromophenols, identified as 1 (3,5-dibromo-2,6-dihydroxyacetophenone), 2 (3-bromo-2,6-dihydroxyacetophenone), and 3 (3,5-dibromo-2,4-dihydroxyacetophenone), were characterized by nuclear magnetic resonance (NMR) spectroscopy. The antibacterial activity against *S. aureus* was evaluated by an agar diffusion method with minimum inhibitory concentration (MIC) values of 24 µg/mL and 12 µg/mL for compounds 1 and 2, respectively. This was comparable to ampicillin and tobramycin, which showed MIC values of 10 µg/mL and 25 µg/mL, respectively. The results demonstrate the antibacterial potential of bromophenol derivatives, especially against antibiotic-resistant strains such as MRSA [98].

Thrombin inhibition has also been reported for the bromophenol derivative (+)-3-(2,3-dibromo-4,5-dihydroxyphenyl)-4-bromo-5,6-dihydroxy-1,3-dihydro-isobenzofuran, isolated as well from *L. nana*, both in vivo and in vitro [94]. Thrombin is an enzyme part of the coagulation cascade, attractive as a target for cardiovascular disease treatment. A novel brominated diphenyl methane derivative, rawsonol, isolated from the green algae *Avrainvillea rawsoni*, has been shown to inhibit the activity of HMG-CoA reductase, a key enzyme in cholesterol biosynthesis. This indicates the potential use of rawsonol as a cholesterol-lowering therapy [99]. These studies are summarized in Table 2.

## 4. Primary Terpenoids: Chlorophylls, Phycobilins, and Carotenoids

### 4.1. Biosynthesis and Structural Characterization of Primary Terpenoids

Terpenoids, also referred to as isoprenoids, are a large and diverse class of naturally occurring organic compounds derived from isoprene units. In macroalgae, terpenoids are classified in two main categories: primary terpenoids and secondary terpenoids [104]. Interest in primary terpenoids (PTs) has increased in recent years due to their role as natural pigments, which have attracted consumer attention as alternatives to synthetic additives. Moreover, these compounds are associated with various bioactive properties, further enhancing their appeal [105].

Macroalgae are recognized as excellent sources of PTs, which encompass three primary pigment types: carotenoids, chlorophylls, and phycobiliproteins, corresponding to Phaeophyta, Chlorophyta, and Rhodophyta [106]. Generally, Chlorophyta (green algae), contain mainly chlorophyll *a* and *b*, as well as carotenoids; Rhodophyta (red algae) contain chlorophyll *a*, phycobiliproteins, and carotenoids; in turn, Phaeophyta (brown algae) contain chlorophyll *a*, *c*, and carotenoids [107].

**Figure 3:**
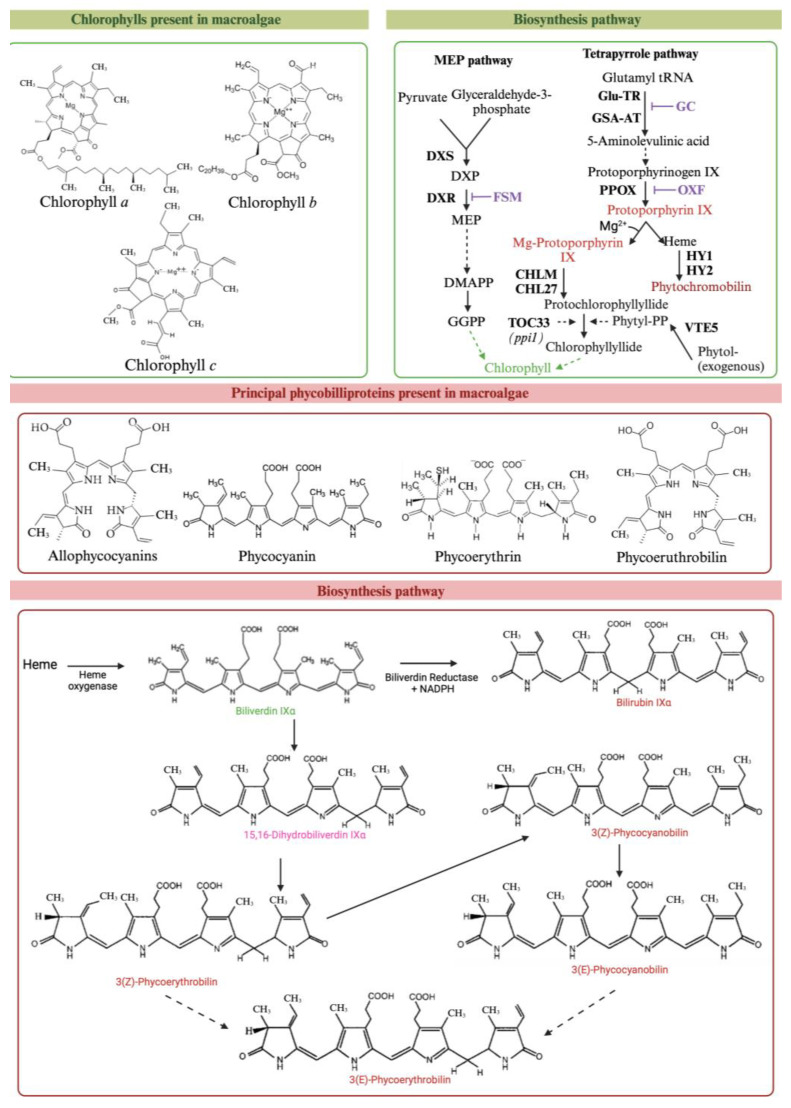
Overview of the biosynthetic pathway of the chlorophylls and phycobilins in macroalgae.

Chlorophylls are the most abundant PTs in nature [108]. These hydrophobic metabolites are able to convert light into biological energy [109,110]. The chlorophyll molecule (with a molar mass of 893.5–925.5 g/mol) is a cyclized chromophore and has four pyrrole rings and a porphyrin ring with a sequestered magnesium atom inside. The ring, a highly chelating ligand, is the main component of the molecule and can be bonded to different substituents, leading to four chlorophyll isoforms, ranging from *a* to *d*: *a*, *b*, and *c* being the most common ones [106,110,111,112]. It is important to note that chlorophyll is also present in charophytes, a group of green algae closely related to land plants, which contain both chlorophyll *a* and chlorophyll *b*. The chlorophyll biosynthesis pathway consists of more than 15 enzymatic reactions. After the stereospecific reduction of the double bond in the D-ring of protochlorophyllide (Pchlide), chlorophyllide (Chlide) is produced and immediately converted to chlorophyll *a* (Figure 3) [113].

The molecular structure of chlorophyll *a* is C_55_H_72_MgN_4_O_5_ and it is the most abundant tetrapyrrole pigment. Its color ranges from blue to green, with a maximum absorption range of 600–665 nm [114]. Chlorophyll *b* (C_55_H_70_MgN_4_O_6_), primarily present in green macroalgae, is a green/yellow pigment with maximum absorption of between 642 and 652 nm. Additionally, chlorophyll *c* has a blue/greenish color and a maximum absorption range of 447 to 452 nm (using acetone as solvent). This isoform includes two types: chlorophyll *c*1 (C_35_H_30_MgN_4_O_5_), with an absorption peak at 447 nm, and chlorophyll *c*2 (C_35_H_28_MgN_4_O_5_), with an absorption peak at 450 nm [106,112]. Chlorophyll *d* (red-shifted chlorophyll), first identified in 1943 in small quantities in red algae, is now recognized as the PT in cyanobacterium and red algae [115]. Chlorophyll *d* (C_54_H_70_MgO_6_N_4_) exhibits three distinct absorption maxima at 696, 456, and 400 nm in methanol [116].

Phycobiliproteins, which are hydrosoluble, non-toxic, fluorescent, and highly stable proteins [106]. From a structural point of view, these pigments consist of protein subunits bonded to bilins (prosthetic group) [117]. These PTs can be classified into four groups: phycoerythrins, allophycocyanins, phycoerythrocyanins, and phycocyanins [106]. The functions of these PTs are direct light absorption and participation in the energy transfer chain with the phycobilisome, which goes from phycoerythrin to phycocyanin, to allophycocyanin, and to chlorophyll *a*, in this order [112]. These PTs (with a maximum absorption range varying from 450 to 570 nm) are highly abundant in Rhodophyta and in cyanobacteria, such as *Arthrospira platensis* [106]. In fact, the red color of these algae is mainly due to the presence of phycoerythrin [112]. For phycobilin biosynthesis, heme groups are formed by the action of heme oxygenase and reduction by ferredoxin-dependent bilin reductase (FDBR), leading to biliverdin IXα (Figure 3) [117].

Carotenoids are yellow to red PTs that are present in a wide range of seaweeds, especially in brown seaweeds. The main function of these compounds is to collect and transfer light energy from chlorophyll [106,112]. Moreover, this highly lipophilic PT has a photoprotective action against photo-oxidative damage [112,118]. The structure of carotenoids is characterized by a symmetrical tetraterpene skeleton with the tail-to-tail linkage of the two C_20_ moieties. In addition, the extensive system of conjugated bonds allows for the absorption of light, resulting in the strong coloring of these molecules [118].

**Figure 4:**
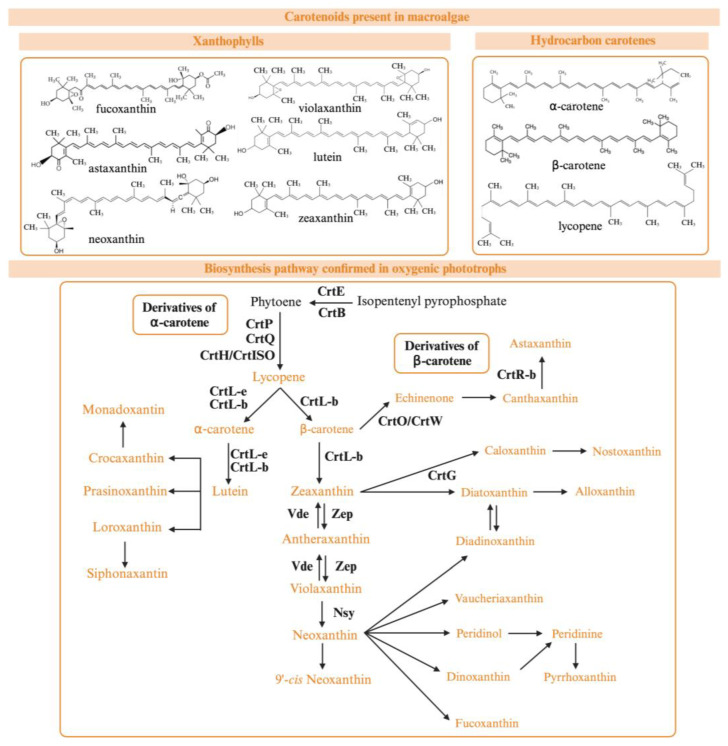
Overview of the biosynthetic pathway of carotenoids in macroalgae and plants. CrtE: geranylgeranyl pyrophosphate synthase; CrtB: phytoene synthase; CrtP: phytoene dehydrogenase; CrtQ: zeta-carotene desaturase; CrtG: carotenoid C2-hydroxylase; CrtH: prolycopene isomerase; CrtISO: carotene cis-trans isomerase; CrtW: β-carotene ketolase; CrtL-e: lycopene ε-cyclase; CrtL-b: lycopene β-cyclase; CrtR-b2: β-carotene hydroxylase; crtO: glycosyl-4,4′-diaponeurosporenoate acyltransferase; Vde: violaxanthin de-epoxidase; Zep: zeaxanthin epoxidase; Nsy: neoxanthin synthase.

Considering their molecular structure, these PTs can be classified in two groups: xanthophylls (astaxanthin, fucoxanthin, loraxanthin, lutein, violaxanthin, zeaxanthin, and neoxanthin), and hydrocarbon carotenes (α-carotene, β-carotene, and lycopene). Xanthophylls are characterized by having oxygen atoms within their structure, while hydrocarbon carotenes lack this atom [106]. Regarding carotenoid biosynthesis (carotenogenesis), it has been mainly studied in cyanobacteria and plants, but algae show some common pathways with these organisms, probably because of their homologous genes (Figure 4). However, other genes and enzymes involved in carotenogenesis are specific to seaweeds, and their roles in carotenogenesis are yet to be determined [113].

### 4.2. Biological Properties of Natural Pigments with Relevance in Disease Treatment

Today, there is an emerging need to exploit natural sources of therapeutic compounds; seaweeds, owing to their high content of bioactive compounds, represent a paradigmatic example of these natural sources [119]. Chlorophylls [120], phycobiliproteins [121], and carotenoids [106], including those in edible algae, have been proven to harbor important biological properties, such as antioxidant, neuroprotective, anti-radical, anti-inflammatory, antimutagenic, antitumor, antiviral, and immunostimulant activities. Among the previous, the PTs from seaweeds have been highlighted for their antioxidant and neuroprotective activities; therefore, these biological properties are discussed below and characterized in Table 3.

#### 4.2.1. Antioxidant Activity of Natural Pigments

Brown algae have shown the highest antioxidant activity, followed by red and green algae [113]. The phycobiliprotein antioxidant ability can be explained by its structure, which is similar to bilirubin, a potent antioxidant and in vivo ROS scavenger [128]. This activity has been evaluated using different methodologies: e.g., the antioxidant activity of phycobiliproteins from *P. yezoensis* was studied by using ROS inhibition assays in HepG2 (human hepatoblastoma cell lines); after the phycobiliprotein synthesis, the results obtained from the ROS tests showed the potential antioxidant activity of these pigments, especially phycoerythrin (PBP2), which downregulated the ROS and appeared to function through p-Nrf2/SOD pathways [123].

The antioxidant activity of carotenoids has been explained by the transfer of the excess energy of singlet oxygen in the long centric chain [113]. Carotenoids are involved in scavenging reactions of the two ROS: singlet molecular oxygen and peroxyl radicals [129]. Thus, carotenoids scavenge oxidizing free radicals via three reactions: electron transfer with a carotenoid radical cation formation, electron transfer with a radical cation formation, and hydrogen atom transfer forming a neutral carotenoid radical [113]. In a study reporting the antioxidant activity of lipophilic extracts from *Himanthalia elongata*, *Laminaria saccharina*, and *L. digitata*, the carotenoid and chlorophyll content ranged from 2.19 to 3.15 μg carotenoids/g and 2.88 to 3.86 μg chlorophyll/g. After the extraction process, the potential radical-scavenging capacity against DPPH radicals achieved half of the maximum effective concentration (EC_50_) 98.3 mg/L. Metal ions were also used to determine the radical-scavenging capacity, achieving an EC_50_ that ranged from 228.6 to 532.4 mg/L. Moreover, the extracts also showed ferric-reducing power, with results ranging from 8.3 to 26.3 mg Trolox equivalents/g. The antioxidant potential of the extracts was linked to the different pigments identified by liquid chromatography, coupled with a photodiode array detector and electrospray ionization tandem mass spectrometry (LC-PDA-ESI-MS/MS), including fucoxanthin, violaxanthin, *β*-carotene, and chlorophyll *a* and *b* [122]. The antioxidant activity of pigments from *Sargassum* sp. was also assessed. Chlorophyll *a*, *b*, and *c*1 + *c*2, fucoxanthin, phycocyanin, and phycoerythrin were the target pigments to determine the antioxidant activity in algae extracts by using DPPH and ferric-reducing antioxidant power (FRAP) assays. The results showed an IC_50_ = 2.684–2.966 mg/mL for the DPPH-radical-scavenging activity and 14.45 µM Fe^2+^/mg for the FRAP assay [124].

#### 4.2.2. Neuroprotective Activity of Natural Pigments

Neurodegenerative diseases are estimated to be the second most common cause of death in the next decades, surpassing cancer. Therefore, the necessity of looking for natural sources of neuroprotective compounds has increased lately [130], and the PTs present in seaweeds have shown great potential.

Regarding carotenoids, fucoxanthin’s neuroprotective activity was studied, showing the potential of this lipophilic compound to reduce the β-amyloid (Aβ) fibrils and oligomer formation in vitro. Aβ is linked to neurotoxicity production in the early stage of Alzheimer’s disease, so the inhibition of Aβ assembly is considered a primary target in Alzheimer’s disease therapy [131]. Fucoxanthin has also been linked to the amelioration of both inflammation and oxidative responses in microglia. For this study, murine BV2 microglia cells were used, and fucoxanthin showed an effective inhibition of Aβ_42_-induced iNOS expression in a concentration-dependent manner. Moreover, fucoxanthin extracts (5, 10, and 50 μM) showed their inhibitive effect against Aβ_42_-induced PGE_2_ production through the downregulation of the COX-2 gene and protein expressions [126]. Another study of the neuroprotective effect of fucoxanthin, isolated from *S. oligocystum*, demonstrated the H_2_O_2_- and Aβ25-35-induced neurotoxic protection in C6 cell lines through the regulation of the gene expression of antioxidant enzymes (CAT and GPx) [125]. The neuroprotective effect of astaxanthin was studied in post-subarachnoid hemorrhage rats by administrating 25 mg/kg or 75 mg/kg of this carotenoid. The results showed that the treatment with 75 mg/kg of astaxanthin downregulated the increased nuclear factor kappa B activity and the expression of inflammatory cytokines, besides ameliorating secondary brain injury cascades, such as blood–brain barrier disruption, neurological dysfunction, or neuronal degeneration [132].

Based on the studies of pigment bioactivities in algae, these compounds show a potential application as antioxidant and neuroprotective agents. Therefore, the use of these metabolites seems to be an alternative for the development of drugs and nutraceuticals to prevent and/or treat chronic diseases such as Alzheimer’s disease or cancer.

## 5. Secondary Terpenoids

### 5.1. Biosynthesis and Structural Characterization of Secondary Terpenoids

Secondary terpenoids are a diverse group of lipophilic compounds that arise from the modification of primary terpenes through complex biochemical processes [133,134]. Unlike primary terpenoids, which are integral to basic metabolic functions and structural roles, secondary terpenoids are primarily involved in ecological interactions and defense mechanisms [135]. These compounds are synthesized in response to environmental stresses, predation, or competition, and often exhibit significant biological activities such as antimicrobial, antifungal, and antioxidant properties [134].

**Figure 5:**
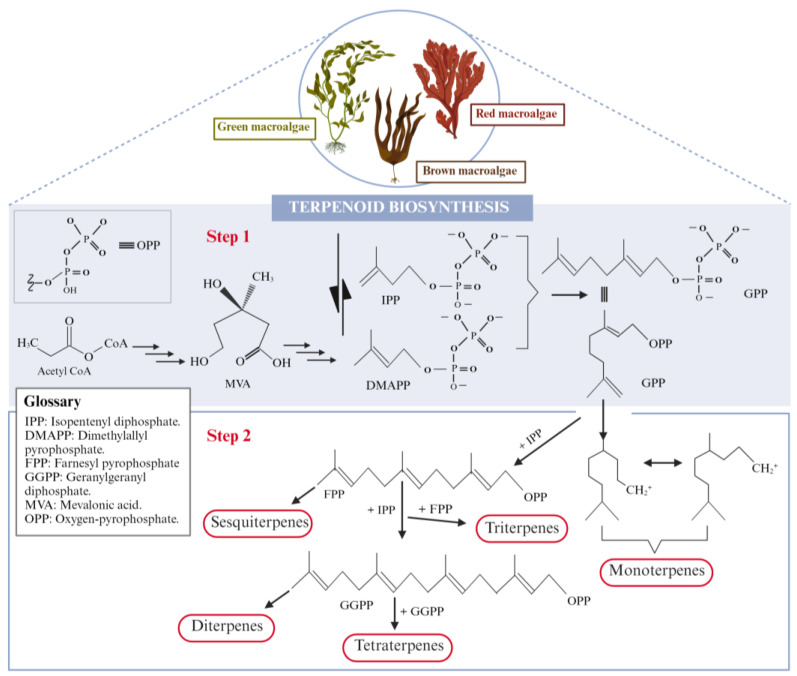
An overview of the biosynthesis of terpenoids in macroalgae.

Marine macroalgae, particularly red seaweeds, are known for their abundance in secondary terpenoids; among their major families, *Rhodomelaceae* stands out as a remarkable source of secondary terpenoids, due to both the quantity present and their chemical diversity (Figure 5) (at least 1058 naturally occurring molecules have been identified and characterized in *Rhodomelaceae*). Remarkably, secondary terpenoids represent approximately 20% of all halogenated compounds in marine organisms [136].

Although red algae are rich in terpene diversity, there is still a significant gap in our understanding of their biosynthesis, in contrast to the well-established knowledge of terpene biosynthesis in land plants [137]; even so, the biosynthetic step characterized by condensing isopentenyl pyrophosphate units is comparable in macroalgae and terrestrial plants’ metabolism [138]. However, in contrast to the mechanisms in land plants, it is supposed that halogens are involved in the biosynthesis of marine terpenes through cyclization reactions. The halogen-induced cyclization of a slightly modified monoterpene precursor yields a halogenoterpene, which can be further halogenated [139]. In either case, terpenoids may be characterized as oxygen-containing derivatives that are polymerized from two or more isoprene (C_5_H_8_) units [18,133,140]; these oxygen-containing derivatives may include alcohols, aldehydes, carboxylic acids, ketones, esters, or glycosides [141]. In strict sense, terpenoids are terpenes that have undergone chemical modifications, such as the incorporation of oxygen at different positions; however, terpenes and terpenoids are often used interchangeably [142].

The secondary terpenoid profile of macroalgae includes several distinct classes, based on their structural skeletons and biosynthetic origins: hemi- (C_5_), mono- (C_10_), sesqui- (C_15_), di- (C_20_), sester- (C_25_), tri- (C_30_), sesquar- (C_35_) and tetraterpenoids (C_40_), consisting mainly of carotenoids that have been combined with the primary terpenoids that were described in the previous section [136,143,144]. In macroalgae and plants, isoprenoids are produced via two distinct biosynthetic pathways: the mevalonate (MVA) pathway and the methylerythritol phosphate (MEP) pathway. The MVA pathway occurs in the cytoplasm and starts with acetyl-CoA, leading to the formation of mevalonic acid (MVA), which serves as the precursor for isopentenyl diphosphate (IPP) and dimethylallyl diphosphate (DMAPP). Meanwhile, the MEP pathway occurs in the plastids and also leads to the formation of IPP and DMAPP, though through a different set of precursors [145].

DMAPP and IPP combine to form geranyl diphosphate (GPP), the precursor for monoterpenes (C_10_). Further additions of IPP result in the formation of farnesyl pyrophosphate (FPP), the precursor for sesquiterpenes (C_15_), and geranylgeranyldiphosphate (GGPP), the precursor for diterpenes (C_20_). Additionally (Figure 5), FPP dimers yield triterpenes (C_30_), while GGPP dimers produce tetraterpenes (C_40_) [141,146,147].

The biological activities associated with some terpenoids present in marine macroalgae have been linked to their lipophilicity and their tendency to partition into cellular membranes, either interacting with membrane-bound proteins or disrupting the membranes’ integrity [148]. In the following sections, the main biological properties of terpenoids are discussed and further summarized in Table 4.

### 5.2. Biological Properties of Terpenoids with Relevance in Disease Treatment

#### 5.2.1. Antioxidant Activity of Terpenoids

The antioxidant potential of macroalgae is generally measured by their $O_{2}^{\cdot -}$, DPPH, and ABTS radical-scavenging activities, total phenolic compounds content in extracts, FRAP, inhibition of lipid peroxidation (e.g., thiobarbituric acid reactive substances formation inhibition), or a Trolox equivalent antioxidant capacity (TEAC) assay. Various reactions and mechanisms, such as the prevention of chain initiation, the binding of transition metal ion catalysts, the reducing capacity, and radical scavenging, have been attributed to validate the antioxidant activity of terpenoids [158]. For instance, fucoxanthin’s antioxidant activity is based on its singlet-oxygen-quenching properties and its free-radical-scavenging ability, which mainly depends on the number of conjugated double bonds and end groups [159]. In a study investigating the antioxidant potential of the halogenated monoterpene 1E,3R,4S,5E,7Z-1-bromo-3,4,8-trichloro-7-(dichloromethyl)-3-methylocta-1,5,7-triene (isolated from *Plocamium* spp., a red macroalga), it showed excellent DPPH-radical-scavenging activity, with an IC_50_ value of 50 ± 10 μM, comparable to the one (20 ± 4 μM) obtained for the positive control ascorbic acid (AsA) [149]. In another study, *Codium tomentosum* (green macroalga) and *Plocamium cartilagineum* (red macroalga) were evaluated for their ability to scavenge $O_{2}^{\cdot -}$ and nitric oxide (NO); the aqueous extracts of the two algae showed concentration-dependent $O_{2}^{\cdot -}$ scavenging, higher in the red algae (IC_25_ = 54 μg/mL) than in the green species (IC_25_ at 66 μg/mL). The analyzed extracts displayed similar protective activity against NO, which was also shown to be concentration dependent: the scavenging ability followed the order *P. cartilagineum* (IC_50_ = 688 μg/mL) > *C. tomentosum* (IC_50_ = 737 μg/mL). Concerning the terpenoid composition, the content of limonene in *C. tomentosum* was particularly noteworthy, being the most abundant compound (28%); three other monoterpenes were also identified (*o*-cymene 0.167%, linalool 3.087% and α-terpineol 1.545%), but in minor quantities. In *P. cartilagineum*, menthone (4.299%) was the only identified terpenoid [138].

#### 5.2.2. Antimicrobial Activity of Terpenoids

Terpenoids are active against microbes through five main mechanisms: (i) cell membrane destruction, (ii) anti-quorum sensing (QS) action, (iii) the inhibition of ATP and its enzymes, (iv) the inhibition of protein synthesis, and (v) a synergistic effect. In such manner, terpenoids disrupt bacterial cell membranes by utilizing their lipophilic nature, penetrating the lipid bilayer and exerting antibacterial or antifungal effects. They also interfere with QS; a communication system used by microbes to coordinate activities and promote antibiotic resistance. Besides, terpenoids inhibit ATP and its enzymes, disrupt protein synthesis, and exhibit a synergistic effect, enhancing their overall antimicrobial activity [133].

In a study screening (by the agar diffusion technique) the antimicrobial activity of haloterpenes from the dichloromethane-methanol extract of *Laurencia papillosa* (red macroalgae) against *Bacillus subtilis*, *Staphylococcus aureus*, *Streptomyces viridochromogenes* Tü 57, *Streptococcus pyogenes*, *Escherichia coli*, *Shigella* sp., *Proteus* sp., *Candida albicans*, *Mucor miehei* Tü 284, and *Chlorella vulgaris*, the main activity was measured against the Gram-positive *S. viridochromogenes* (30 mm at 400 µg/disc). Among the isolated compounds, aplysiolic acid and aplysiol-7-one were weakly active against *S. aureus* (10.5 mm at 40 µg/disc) [151]. In a related antimicrobial study, the results showed that the ethanolic extracts of two marine macroalgae (*Laurencia paniculata* and *Ulva prolifera*) were active against various fungal pathogens with minimum inhibitory concentrations (MIC) and minimum fungicidal concentrations (MFC) in the range of 125 and 500 µg/mL. A gas chromatography–mass spectrometry/tandem mass spectrometry (GC-MS/MS) analysis revealed the presence of terpenes, terpene alcohols, steroids, and sesquiterpenes in the macroalgal extracts, suggesting their potential in combating fungal infections and aiding in the treatment of chronic bronchial asthma [152].

#### 5.2.3. Anti-Inflammatory Properties of Terpenoids

Terpenoids, such as andrographolide and triptolide, work by targeting specific pathways in the body associated with inflammation [160]. Studies have shown that these terpenoids can inhibit the expression of ICAM-1, a signaling receptor in many cell types to mount inflammatory responses, and reduce the synthesis of NO in human cells, which in turn decreases NO production, an important signaling mediator that plays a key role in the pathogenesis of inflammation [161,162]. Additionally, triptolide has been found to inhibit the NF-κB-dependent transcription, thereby suppressing the production of pro-inflammatory cytokines such as TNF-α and IL-6 in inflamed cells. By targeting these pathways, terpenoids help to alleviate inflammation, making them potential treatments for chronic autoimmune disease like rheumatoid arthritis [163]. Yet, it is essential to consider that some terpenes may face hindrances. For instance, they might exhibit toxicity or have poor water solubility, which can restrict their efficacy and safety in clinical use. Therefore, while terpenoids reveal promise as anti-inflammatory agents, the careful consideration of their side effects is necessary for their practical application [160,164]. Among the anti-inflammatory terpenoids of macroalgae, fucosterol, epitaondiol, neorogioltriol, pacifenol, ergosterol, 7-dehydroporiferasterol, pheophytin A, and apo-90-fucoxanthinone are the most commonly isolated compounds [165]. For example, in a study aimed at investigating the anti-inflammatory effects of apo-9′-fucoxanthinone in LPS-stimulated murine macrophage (RAW264.7) cell lines and zebrafish models, it was concluded that fucoxanthinone significantly reduced the NO and PGE2 production, as well as inducible nitric oxide synthase (iNOS) and COX-2 expression in the LPS-induced RAW264.7 cells. It also decreased (in a dose-dependent manner) the pro-inflammatory cytokines TNF-α, IL-1β, and IL-6. Mechanistically, fucoxanthinone inhibited the MAPKs (p38, JNK, ERK) and NF-κB pathways, resulting in decreased iNOS and COX-2 expression. In zebrafish, fucoxanthinone suppressed ROS and NO production in a similar way, as well as suppressing the expression of iNOS, COX-2, TNF-α, and IL-1β. These results suggest that fucoxanthinone may be a potent anti-inflammatory agent, with possible applications as a nutraceutical or in the pharmaceutical industry [154].

In another study, fucosterol, isolated from *Padina boryana*, was evaluated against particulate-matter-induced inflammation in RAW 264.7 macrophages. Fucosterol effectively inhibited NO under the PM-stimulated conditions and downregulated (in a dose-dependent manner) inflammatory mediators, such as iNOS, COX-2, PGE2, and the pro-inflammatory cytokines IL-1β, IL-6, and TNF-α. Additionally, fucosterol’s effects were strengthened by suppressing the MAPK and NF-κB pathways. The Nrf2/HO-1 pathway indicated a reduction in ROS due to fucosterol’s activity, suggesting that it could protect against PM-induced inflammation [153].

## 6. Conclusions

In conclusion, phlorotannins, bromophenols, natural pigments, and terpenes are prominent bioactive compounds found in marine macroalgae. Phlorotannins, polyphenols, and bromophenols (all with a wide range of structures), exhibit a variety of beneficial properties, including antioxidant, antitumor, or antiviral properties. Although the synthesis and properties of these compounds are not yet fully understood, they represent promising sources of bioactive compounds with potential applications in the pharmaceutical and cosmetic industries. On the other hand, marine macroalgae are valuable sources of natural pigments such as carotenoids, chlorophylls, and phycobiliproteins, which also exhibit a wide range of bioactive properties, including antioxidant, anti-inflammatory, and neuroprotective effects. Their therapeutic potential places them as promising candidates for the development of drugs and nutraceuticals targeting metabolic disorders such as diabetes and neurological disorders like Alzheimer’s disease. Finally, terpenoids found in marine algae, especially in red algae, such as those of the Rhodomelaceae family, exhibit a wide variety of biological properties such as antioxidant, antimicrobial, and anti-inflammatory effects. Although the biosynthetic pathways associated with these compounds are not completely understood in algae, their therapeutic potential may highlight these species as promising candidates for the development of drugs and nutraceuticals against various diseases. In the future, research is expected to continue to deepen our understanding of these compounds and further explore their therapeutic applications, which could open new opportunities in the fields of medicine and marine biotechnology.

## Figures and Tables

**Table 2:** Macroalgal bromophenols properties and activity results.

Properties	Active compound	Species	Concentration	Assay	Results	Ref.
Antioxidant	C1	*Vertebrata lanosa* (R)	nd	DPPH	IC_50_ = 7.43 µM	[85]
Antioxidant	Nitrogen-containing bromophenols	*Rhodomela confervoides* (R)	nd	DPPH	IC_50_ = 5.22 µM	[90]
			nd	ABTS (TEAC)	2.87 µM	
Anti-obesity	Red algae bromophenol extract	*Rhodomelaceae* (R)	100 μg/mL	G6PD inhibition in vitro	IC_50_ = 0.85 µM	[92]
Antidiabetic	C2	*Rhodomela confervoides* (R)	nd	DPPH	IC_50_ = 8.28 µM	[52]
Anti-cancer	C3	*Rhodomela confervoides* (R)	140 μg/mL	In vitro cytotoxicity against BEL7402	3.18 µg/mL	[96]
Anti-cancer	C4	*Leathesia nana* (B)	0.1%	In vitro cytotoxicity against BEL7402	0.0019 µg/mL	[97]
Anti-inflammatory	Vidalols A and B	*Vidalia obtusaloba* (R)	1.235 g/kg fw and 54 mg/kg fw	Inhibition of PLA2 in vitro	50 µg in agar plate	[100]
Anti-inflammatory	BBDE	*Polysiphonia morrowii* (R)	2 μM	In vitro suppression of LPS induced ROS generation on RAW 264.7 cells	NS	[101]
Antioxidant, anti-inflammatory	Lanosol isopropyl ether, Bromourceolatols A-G	*Ceramium* sp. (R)	25 μg/mL		32 µM	[102]
Anti-Alzheimer’s, antidiabetic	C4	*Symphyocladia latiuscula* (R)	nd	Inhibition of amyloid plaque aggregation	IC_50_ = 20 µM	[103]
Antimicrobial, antifungal	C5	*Odonthalia corymbifera* (R)	nd	Against *S. aureus*	IC_50_ = 1.56 µg/mL	[86]
			nd	Against *C. albicanis*		
Antiviral	C6	*Polysiphonia morrowii* (R)	80%	Against INHV	EC_50_ = 19.0 µg/mL	[91]
				Against IPNV	EC_50_ = 8.0 µg/mL	
Anti-stroke, cardiovascular inflammation	C7	*Leathesia nana* (B)	nd	In vitro inhibition of PTP1B	IC_50_ = 0.84 µmol/L	[94]
Inhibition of cholesterol biosynthesis	Rawsonol	*Avrainvillea rawsoni* (B)	0.01% dw	In vitro inhibition of IMPDH	IC_50_ = 7.4 µM	[99]

Abbreviations: IC_50_: half maximal inhibitory concentration; EC_50_: half maximal effective concentration; C1: 2,2′,3-tribromo-3′,4,4′,5-tetrahydroxy-6′-hydroxymethyldiphenylmethane; C2: 2,2′,3,3′-tetrabromo-4,4′,5,5′-tetrahydroxydiphenylmethane, 3-bromo-4,5-bis(2,3-dibromo-4,5-dihydroxybenzyl) pyrocatechol, bis(2,3-dibromo-4,5-dihydroxybenzyl) ether, 2,2′,3-tribromo-3′,4,4′,5-tetrahydroxy-6′-ethyloxymethyldiphenylmethane; C3: bromophenols, 3-bromo-4,5-dihydroxy benzoic acid methyl ester, 3-bromo-4,5-dihydroxybenzaldehyde; C4: 6-(2,3-dibromo-4,5-dihydroxybenzyl)-2,3-dibromo-4,5-dihydroxy benzyl methyl ether; C5: 2,3,6-tribromo-4,5-dihydroxybenzyl alcohol, 2,3,6-tribromo-4,5-dihydroxybenzyl methyl ether, bis-(2,3,6-tribromo-4,5-dihydroxybenzyl) ether; C6: 3,3′,5,5′-tetrabromo-2,2′,4,4′-tetrahydroxydiphenylmethane, 3,3′-dibromo-6,6′-dihydroxydiphenylmethane, 3,3′,5,5′-tetrabromo-6,6′-dihydroxydiphenylmethane; C7: 3-bromo-4,5-dihydroxybenzyl methyl ether, 3-bromo-4,5-dihydroxybenzaldehyde; C8: bromophenol derivative (+)-3-(2,3-dibromo-4,5-dihydroxyphenyl)-4-bromo-5,6-dihydroxy-1,3-dihydro-isobenzofuran; BBDE: Bis (3-bromo-4,5-dihydroxybenzyl) ether; TEAC: trolox equivalent antioxidant capacity; PLA2: phospholipase A2 enzyme; G6PD: glucose-6-phosphate dehydrogenase; BEL7402: cellosaurus cell line BEL-7402, a HeLa derivative; LPS: bacterial lipopolysaccharides; RAW 264.7: macrophage cell line; ROS: reactive oxygen species; INHV: viral hemorrhagic septicemia virus; IPNV: infectious pancreatic necrosis virus; PTP1B: protein-tyrosine phosphatase 1B; IMPDH: inosine-5′-monophosphate dehydrogenase; *S. aureus*: *Staphylococcus aureus*; *C. albicans*: *Candida albicans.* NS: not specified; (G): green macroalgae; (R): red macroalgae; (B): brown macroalgae.

**Table 3:** Macroalgal pigments properties and activity results.

Properties	Active Compound	Species	Concentration	Assay	Results	Ref.
Antioxidant	Lipophilic extract	*Hemipristis elongata*, *Laminaria digitata*, and *Saccharina latissima* (B)	nd	DPPH	EC_50_ = 98.3 mg/L	[122]
			nd	Metal ions	EC_50_ = 228.6–532.4 mg/L	
			nd	FRAP	8.3–26.3 mg Trolox eq/g dw	
Antioxidant	Phycobiliproteins	*Pyropia yezoensis* (R)	10 µg/mL	ROS	Viable cells increased by 23–32%	[123]
Antioxidant	Chlorophyll, fucoxanthin, carotenoid, phycocyanin, phycoerythrin	*Sargassum* sp. (B)	Chl 0.11 mg/g, Fu 0.04 mg/g, Ca 19.5 mg/g	DPPH	IC_50_ = 2.584–2.966 mg/mL	[124]
		*Sargassum olygocystum* (B)		FRAP	9.09–14.45 µM Fe^2+^/mg extract	
Antioxidant	Fucoxanthin	*Sargassum olygocystum* (B)	2.9 mg/g dw	DPPH	IC_50_ = 3.42 mg/mL	[125]
Neuroprotective	Fucoxanthin	NS	nd	PGE_2_ production	MEI = 2034.2 pg/mL	[126]
Neuroprotective	Fucoxanthin	*Sargassum olygocystum* (B)	2.9 mg/g dw	MTT assay	Viable cells increased by 91.23–97.69%	[125]
Neuroprotective	Astaxanthin	*Acetabularia acetabulum* (G)	nd	MTT assay	10,000 µM	[127]

Abbreviations: nd: not determined; Chl: chlorophyll a; Fu: fucoxanthin; Ca: carotenoid; IC_50_: half maximal inhibitory concentration; EC_50_: half maximal effective concentration; DPPH: 2,2′-diphenyl-1-picrylhydrazyl radical; FRAP: ferric-reducing antioxidant power; ROS: reactive oxygen species; MEI: minimum effective inhibition; PGE2: prostaglandin-E2 production; MTT: 3-[4,5-dimethylthiazol-2-yl]-2,5 diphenyl tetrazolium bromide. NS: not specified; (G): green macroalgae; (R): red macroalgae; (B): brown macroalgae.

**Table 4:** Macroalgal terpenoids properties and activity results.

Properties	Active Compound	Species	Concentration	Assay	Results	Ref.
Antioxidant	Halogenated monoterpene ^(1)^	*Plocamium* spp. (R)	nd	DPPH	IC_50_ = 50 μM; AsA: IC_50_ = 20 μM	[149]
Antioxidant	Limonene and linalool	*Codium tomentosum* (G)	28.1 and 3.1%	$O_{2}^{\cdot -}$	IC_25_ = 66 μg/mL	[138]
	Menthone (monoterpene)	*Plocamium cartilagineum* (R)	4.3%		IC_25_ = 54 μg/mL	
Antioxidant	Sesquiterpenes	*Ulva fasciata* (G)	nd	DPPH, ABTS^+^	DPPH (89.8%), ABTS^+^ (82.6%)	[150]
Antibacterial	Haloterpenes (aplysiolic acid)	*Laurencia papillosa* (R)	nd	ADT	*S. aureus* (10.5 mm)	[151]
Antifungal	Diterpenes and sesquiterpenes	*Laurencia paniculata* (R), *Ulva prolifera* (G)	0.18–13.91%	MIC and MFC	*C. albicans* (MIC = 125 µg/mL; MFC = 125 µg/mL)	[152]
Anti-inflammatory	Fucosterol (diterpenoid)	*Padina boryana* (B)	125 µg/mL	NO Inhibition	Suppressed the expression of iNOS, COX-2, and PGE2	[153]
Anti-inflammatory	Apo-9-fucoxanthinone (carotenoid)	NS	25–100 μg/mL	In vivo zebrafish model	Downregulated iNOS, COX-2, TNF-α, and IL-1β	[154]
Cytotoxicity	Terpenoid ^(1)^	*Gracillaria dura* (R)	nd	MTT assay	CC_50_ (Vero cells = 6; Ribavirin = 2.5 mg/mL);	[155]
Cytotoxicity	Halogenated monoterpenes	*Plocamium cartilagineum* (R)	nd	MTT assay	MIC (SW480 cells = 131; Lindane > 344)	[156]
Cytotoxicity	Brassicolene (diterpenoid)	*Gelidium latifolium* (R)	107.06 mg GAE/g	MTT assay	CC_50_ (B16-F10 cells = 84.29 µg/mL)	[157]

Abbreviations: nd: not determined; DPPH: 2,2-diphenyl-1-picryl-hydrazyl radical; AsA: ascorbic acid; $O_{2}^{\cdot -}$: superoxide-radical-scavenging activity; ABTS^+^: 2,20-azino-bis(3-ethylbenzothiazoline-6-sulfonate)-scavenging assay; *S. viridochromogenes*: *Streptomyces viridochromogenes* Tü 57; IC_25_: inhibitory concentration 25%; IC_50_: half maximal inhibitory concentration; CC_50_: 50% of the concentration that reduces cell viability by 50% compared to control; ADT: agar diffusion test; MFC: minimum fungicidal concentration; MIC: minimum inhibitory concentration; *C. albicans*: *Candida albicans;* NO: nitric oxide; iNOS: inducible nitric oxide synthase; COX-2: cyclooxygenase-2; PGE2: prostaglandin E2; TNF-α: tumor necrosis factor-α; IL-1β: interleukin-1 β; SW480: human colon adenocarcinoma cell line; MTT: (3-[4,5-dimethylthiazol-2-yl]-2,5 diphenyl tetrazolium bromide); B16-F10: murine melanoma cell line. ^(1)^ 1E,3R,4S,5E,7Z-1-bromo3,4,8-trichloro-7-(dichloromethyl)-3-methylocta-1,5,7-triene. NS: not specified. (G): green macroalgae; (R): red macroalgae; (B): brown macroalgae.

## Data Availability

Not applicable.
